# Technical Improvement and Application of Hydrodynamic Gene Delivery in Study of Liver Diseases

**DOI:** 10.3389/fphar.2017.00591

**Published:** 2017-08-30

**Authors:** Mei Huang, Rui Sun, Qiang Huang, Zhigang Tian

**Affiliations:** ^1^Anhui Province Key Laboratory of Hepatopancreatobiliary Surgery, Department of General Surgery, Anhui Provincial Hospital Affiliated with Anhui Medical University Hefei, China; ^2^Institute of Immunology, School of Life Sciences and Medical Center, University of Science and Technology of China Hefei, China

**Keywords:** hydrodynamic injection, gene delivery, gene therapy, liver diseases, HBV

## Abstract

Development of an safe and efficient *in vivo* gene delivery method is indispensable for molecular biology research and the progress in the following gene therapy. Over the past few years, hydrodynamic gene delivery (HGD) with naked DNA has drawn increasing interest in both research and potential clinic applications due to its high efficiency and low risk in triggering immune responses and carcinogenesis in comparison to viral vectors. This method, involving intravenous injection (i.v.) of massive DNA in a short duration, gives a transient but high *in vivo* gene expression especially in the liver of small animals. In addition to DNA, it has also been shown to deliver other substance such as RNA, proteins, synthetic small compounds and even viruses *in vivo*. Given its ability to robustly mimic *in vivo* hepatitis B virus (HBV) production in liver, HGD has become a fundamental and important technology on HBV studies in our group and many other groups. Recently, there have been interesting reports about the applications and further improvement of this technology in other liver research. Here, we review the principle, safety, current application and development of hydrodynamic delivery in liver disease studies, and discuss its future prospects, clinical potential and challenges.

## Introduction

Gene over-expression or knockdown in cells or tissues is the primary means for gene function study and even gene therapy. It involves various approaches, including viral vectors, lipids, polymers, microinjection, electroporation and so on. Viral vector-mediated gene delivery exhibits the most effective therapeutic results, including adenovirus (Pjechova et al., 2015), adeno-associated virus (Duan, 2016), retrovirus (Doi and Takeuchi, 2015; Vargas et al., 2016), lentivirus (Levy et al., 2015), herpes virus (Wang and Liao, 2017), Epstein-Barr virus (Li et al., 2016) and so on (Oehmig et al., 2004; Nayerossadat et al., 2012; Kotterman et al., 2015; Lukashev and Zamyatnin, 2016). However, its application *in vivo* as a standard treatment is still controversial, due to the lethal immune response (Reid et al., 2002) and carcinogenesis (Hacein-Bey-Abina et al., 2003) caused by the virus vector. In contrast, other gene delivery methods that do not involve viruses are being extensively studied for use of clinical application. The most popular method is plasmid DNA (pDNA)-based gene delivery methods, due to its advantage in safety and versatility *in vivo*. However, its gene transfer efficiency is much lower and restricted to injection site which limits its applications. To achieve better gene-delivery efficiency while maintaining safety features, hydrodynamic gene delivery (HGD), a new gene delivery method has been developed in recent years.

HGD generated hydrodynamic pressure by rapid injection of large doses of DNA liquid into the incompressible blood in circulation, which then permeated the parenchymal cells to allow DNA entry. Hydrodynamics-mediated non-viral vector delivery, due to its high efficiency of gene transfer, significantly reduces DNA degradation by serum and cellular DNase reported in the conventional intravenous injection. Thus, it has been widely employed to facilitate the intracellular delivery of DNA, RNA (Giladi et al., 2003; Kang et al., 2010), proteins (Zhang et al., 2004), polymers (Viegas et al., 2011), and other compounds (Zhang et al., 2004). Recently, HGD has also been used to deliver viral vectors for improving the transduction efficiency (Condiotti et al., 2004; Arad et al., 2005; Brunetti-Pierri et al., 2005; Fujita et al., 2006).

Gene expression *in vivo* led by HGD with pDNA has been shown to be long-lasting (~4 weeks) (Zhang et al., 2000; Miao et al., 2001; Stoll et al., 2001; Yew et al., 2001; Alino et al., 2003; Chen et al., 2003; Score et al., 2006) and can reach therapeutic levels (Kishida et al., 2003; Ye et al., 2003; Held et al., 2005). Ninety percent of products of transgenes by HGD are mainly found in the liver which makes HGD more preferential for liver specific studies. Low levels of expression can be also detected in the spleen, heart, kidneys and lungs (Liu et al., 1999; Hamar et al., 2004). HGD has recently been shown to mediate a systemic human cytokine expression in humanized mouse model. A single injection of cytokine expressing plasmids by this procedure led to transgene for more than a month which improved human hematopoietic cell functions in all the organs (Chen et al., 2009, 2015; Li et al., 2013; Yong et al., 2016). Despite a few applications in systemic soluble factor expression, HGD is mainly reported for gene delivery into the liver and further study of hepatotropic diseases.

The liver, as one of the largest metabolic organs, is a target of various viral infections. Meanwhile, it is also an immune tolerant organ responsible for the synthesis of complement components, acute phase proteins and cytokines (Bandyopadhyay et al., 2016; Gao, 2016; Grakoui and Crispe, 2016; Horst et al., 2016; Peng et al., 2016; Robinson et al., 2016; Zhou et al., 2016). Therefore, the disorder of these gene products, either deficiency or over-expression, can result in anomaly of immune regulation and the occurrence of liver disease (Ju and Tacke, 2016). As a liver-targeted gene delivery method, HGD shines the study of liver diseases. In the following summary, we first review the principle, efficiency and safety of the experimental procedures of HGD. Subsequently, the current various applications of HGD in liver disease study as well as the future perspectives of the hydrodynamics-based procedure is discussed.

## The principle and safety of the liver targeted gene delivery method

A successful HGD requires a rapid (5–7 s) intravenous injection of solution in a high dose (7–10% of body weight) (Liu et al., 1999). The large amount of solution rapidly flows into the vena cava, and then reflux, because it cannot be rapidly pumped by the heart. The liver collects most of the reflux fluid and takes up the bulk of the injected pDNA solution, with larger vessels to the central vessel than other organs. The accompanying increased pressure enhances permeability of parenchymal cells (hepatocytes in liver) and transiently generates numbers of membrane pores to allow pDNA entry and gene expression (Zhang et al., 2004).

Green fluorescence protein (GFP) has been used to localize the expression of transgenes by HGD. Abundant GFP expression was mainly found in liver after GFP expression plasmid delivery by HGD, while the signals in other organs were very weak (Crespo et al., 2005). This confirms HGD mainly results in expression in the liver. Actually it has been calculated that ~40% of hepatocytes are transfected when a dose of 10 μg plasmid DNA is administrated into mouse tail vein (Liu et al., 1999). Similar distribution of expression and transfection efficiency were also observed in chickens (Hen et al., 2006).

Various substances can be delivered by HGD, and it is considered as a pressure-mediated delivery method, without involvement of receptor-ligand interaction. Concerning HGD's safety, it has been shown that the transient high pressure caused by hydrodynamic injection results in a temporary cardiac dysfunction, a sharp rise of venous pressure, liver congestion, and elevation of aminotransferase (ALT). However, the transient abnormal ALT usually recovers within 2–3 days post-injection with no signs of hepatic failure (Zhang et al., 2004). In general, the negative impact of hydrodynamic injection to the liver very little and well tolerated. Nevertheless, HGD will not be directly applied to the human body in this way. Dogs, pigs and other large animals are being used as the main animal models of the pre-clinical trials to try HGD's optimal delivery method (Kamimura et al., 2014; Stoller et al., 2015; Yokoo et al., 2015; Hyland et al., 2017).

## Regulation of gene expression in liver by HGD

HGD, as an efficient means of regulating gene and protein expression in liver, has become an important means of studying the relevance of proteins of interest to various liver diseases, as well as some systemic diseases that can be treated by altering gene expression in liver. Gene regulation, including gene over-expression and/or gene knock-down, can not only be used for gene function study, but also as a means of gene therapy for liver diseases.

### Gene over-expression in liver

As one of the most effective methods for *in vivo* delivery of pDNA, HGD was first widely employed for liver targeted gene over-expression. Among the various genes of interests, cytokines are usually over-expressed by HGD in gene therapy application. Therapeutic effect of interferon (IFN)-γ by HGD of pDNA has been demonstrated in murine liver and lung metastasis models (Kobayashi et al., 2002). IL-22 over-expression in the liver induced by HGD of IL-22 cDNA expressing plasmid protected the liver from various toxins induced damage, which indicates that IL-22 has the therapeutic potential in the treatment of human liver disease (Pan et al., 2004). Hydrodynamic injection of IL-15 expressing plasmid (pLIVE-IL-15) induced sustained high expression of IL-15 in serum and liver of mice, and broke HBV-induced immunotolerance (Yin et al., 2012). In addition, many other secreted proteins have been introduced in liver by this effective method, for determination of their functions and evaluation of their therapeutic activities, including IL-10 (Hong et al., 2003), IL-21 (Brady et al., 2004), hepatocyte growth factor (Yang et al., 2001), human growth hormone (Sondergaard et al., 2003), human factor IX (Miao et al., 2001; Ye et al., 2003), activin (Chabicovsky et al., 2003), human alpha-1 antitrypsin (Zhang et al., 2000), Erythropoietin receptor-IgG1Fc fusion protein (Maruyama et al., 2004).

However, a number of reports have recently shown that multiple genes co-expression may be more effective than single gene over-expression. Over-expression of IL-12 or GM-CSF alone induced by hydrodynamic injection are able to produce strong antitumor cellular immunity, while combined HGD of the two cytokine yielded better effect and achieved more pronounced therapeutic efficacy (Wang et al., 2001).

### Gene knock-down in liver

In addition to gene over-expression, HGD has also been reported as a powerful tool for gene knockdown. Specific gene knockdown by RNA interference was performed mainly in two ways, one is siRNA direct transfection, the other is short hairpin RNA (shRNA) carrying DNA vector transfection. The latter generated siRNA *in vivo*. However, due to the lack of efficient delivery methods for siRNA or shRNA vector, RNA interference is limited *in vivo*.

Researchers silenced *in vivo* Fas expression via hydrodynamic injection of synthesized siRNAs specific for Fas, and protected mice from renal ischemia-reperfusion injury or fulminant hepatitis (Song et al., 2003; Hamar et al., 2004). A similar strategy was applied to suppress endogenous mdr1a/1b gene expression, an efflux pump for various drugs, by HGD of synthetic siRNA or siRNA-expressing pDNA (Matsui et al., 2005). Researcher also used the hydrodynamic approach to co-deliver an optimized siRNA expression construct together with its target reporter gene to demonstrate long-term RNA interference *in vivo* (Chu et al., 2005; Wooddell et al., 2005). Despite all these successful studies of *in vivo* gene knock-down by HGD of siRNA, the RNAi efficiency still remained less optimal. Recently, siRNA-expressing adenoviral vectors were applied by hydrodynamic technique to significantly improve the knock-down effects of gene expression in the murine liver. In this study, adenovirus bearing small hairpin RNA (shRNA) targeting CX3CL1 markedly inhibited CXCL1 expression in the infected livers and reduced adenovirus-induced liver injury (Chen Q. et al., 2008). Concurrently, we have also successfully developed a single vector to target multiple genes at one time: a plasmid containing three shRNA sequences against different NKG2D ligands, which efficiently suppressed NK cell-mediated fulminant hepatitis in mice when delivered by HGD (Huang et al., 2013). Small interfering RNA (siRNA) or siRNA-expressing vectors are the most commonly used tools by HGD to reduce specific gene expression in liver and even intervene viral infection and cancer progression.

### Simultaneous gene over-expression and knock-down in liver

Along with the development of new RNA interference constructs, hydrodynamic injection mediated RNA interference becomes more efficient. Interest has been raised to develop new vectors with more multifunctions. A plasmid vector using an effective liver cell-specific promoter (α-foetoprotein (AFP) enhancer/albumin promoter) was developed to simultaneously over-express and knock-down different genes in a liver-directed manner. This vector has been shown to simultaneous over-expression of human IL-10 and knock-down of endogenous CCL5 and CX3CL1 highly attenuated adenovirus-mediated acute liver injury by inhibiting NK cell recruitment and activation and inflammatory cytokines production (Geng et al., 2013). The escalation and optimization of pDNA vector may reduce the insecurity of HGD by its versatility, which may accelerate the clinical application of HGD to a certain extent.

## Applications of HGD in liver gene therapy

The regulation of liver-target gene expression is closely related with liver-target gene therapy. HGD mediated continuous expression of phenylalanine hydroxylase (PAH) in the mouse liver, played therapeutic roles in the mouse with human phenylketonuria characteristics, by adjusting the level of phenylalanine in blood (Grisch-Chan et al., 2017). Liver targeted expression of circadian clock gene via HGD can relieve metabolic disorders and biological clock disturbances caused by high-fat diet, which may become a treatment for obesity and metabolic disorders in the future (Meyer-Kovac et al., 2017). As one of the most effective delivery methods, hydrodynamic injection also outstands in treatment of liver fibrosis. It has been demonstrated fibrosis in liver affects gene delivery efficiency. HGD of matrix metalloproteinase-13 gene reduced liver fibrosis and showed mentionable gene therapy efficiency (Kobayashi et al., 2016).

## Establishment of viral hepatitis models by HGD

Another major application of hydrodynamic delivery methods is to establish liver disease models for animals, such as viral hepatitis model by over-expressing viral DNA. The most reported work was to model hepatitis B in mouse, including both acute and chronic hepatitis B (Chang et al., 2003; Giladi et al., 2003; Ketzinel-Gilad et al., 2006).

### Mouse model of acute hepatitis B

Although HBV-transgenic mice, which was reported to persistently express distinct HBV antigens or produce infectious virions (Chisari et al., 1987; Wirth et al., 1995; Schirmbeck et al., 2000), have been used to study HBV immunopathogenesis and test numerous drugs for HBV infection, they are inherently tolerant to transgene products (Araki et al., 1989; Moriyama et al., 1990; Guidotti et al., 1995; Larkin et al., 1999; Wang et al., 2016). Therefore, the model is not suitable for studying HBV vaccines. In addition, it is difficult to monitor viral clearance in this model because the HBV DNA fragments are integrated into the genome of every cell (Loirat et al., 2003). Therefore, it is imperative to establish an alternative model of hepatitis B in non-transgenic mice. Hydrodynamic technique was used to deliver the HBV genome coding plasmids into the tail vein.

pAAV-HBV1.2 plasmid, an adeno-associated virus vector (AAV) carrying over-length, replication-competent HBV DNA, was hydrodynamically injected into mice to establish a mouse model mimicking acute HBV infection in human (Zheng et al., 2013, 2016). With the promotion of HGD method, hepatocytes synthesized viral antigens and replicative intermediates and secreted viral particles into the blood circulation. Along with the production of antiviral antibodies, viral antigens gradually disappeared from the blood. So far, there are many reports of acute hepatitis B models established with other HBV genome expressing plasmids in HGD method (Yang et al., 2002; Suzuki et al., 2003). These HGD-based established hepatitis B models provide an excellent platform for studying the pathogenesis of hepatitis B virus and its immune response.

### Mouse model of chronic hepatitis B

However, these animal model of acute hepatitis B could not be applied to chronic hepatitis B studies, which is more popular in the clinic. A mouse model of chronic hepatitis B is urgently needed to explore the chronic pathogenesis of HBV and the immune response. The HBV chronic infection mouse models were also developed by HGD of the HBV genome coding plasmids into tail vein, with a less plasmid concentration than that for acute infection model (Huang et al., 2006; Ju et al., 2009).

After lower concentration of HBV genome expressing plasmids delivery by HGD, immune tolerance occur in 40% of the injected mice, which showed sustained expression of HBV surface antigen for 6 months. The HBV carrier mice also expressed hepatitis B e antigen (HBeAg) and core antigen (HBcAg). Meanwhile, there is a high level of HBV DNA in serum, but along with normal levels of serum alanine aminotransferase, suggesting no significant inflammation of the liver. This is similar to the human chronic HBV infection in the immune tolerance phase (Chen, 1993; McMahon, 2004).

The chronic hepatitis B mouse model helps to better explore liver tolerance during HBV persistence, and test new therapies for chronic HBV infection. With the well-established HBV-carrier mice, researchers have developed many studies on immunpathogenesis and clearance of HBV. Our previous studies demonstrated that KCs supported liver tolerance by inducing IL-10-dependent anti-HBV CD8^+^ T cell exhaustion after HBcAg stimulation (Xu et al., 2014; Li et al., 2015). Meanwhile, γδ T cells drove myeloid-derived suppressor cell (MDSC)-mediated CD8^+^ T cell exhaustion (Xu et al., 2013; Kong et al., 2014). In addition, the intestinal microflora is critical to the immune responses in the liver, which may determines hepatitis B virus clearance or persistence (Chou et al., 2015). This animal model accelerates mechanistic studies of human chronic hepatitis B infection.

Nevertheless, HBV is not capable to infect mice to mimic HBV infection in humans, thus there are still some limitations of these HBV-carrier mouse models, such as the lack of liver HBV infection (e.g., no infectious particle entry, no delivery between hepatocytes, no covalently closed circular DNA) and possible transient heart and liver trauma resulting from hydrodynamic injection. A chronic hepatitis B model established with adenovirus vector which carried the HBV genome still cannot break these limitations (Huang et al., 2012). Apparently new improvements are desired to advance the study of hepatitis B, possibly with the technology of HGD. Most recently, researchers had upgraded the chronic hepatitis B model by hydrodynamic delivery of HBV precursor recombinant covalently closed circular DNA (prcccDNA). In this model, the HBV genome exist as cccDNA, rather than a linear replicon, which could be replicated effectively and thus established HBV persistence (Qi et al., 2014). There will be more and better plasmids, equipped with HGD ride to establish more practical model of hepatitis B.

### Mouse model of hepatitis C and D

Similarly, other viral hepatitis models may be created by HGD injection of corresponding genomic expression cassettes. In addition to hepatitis B virus, there are other hepatitis viruses that are difficult to establish mouse infection model, due to absence of matched receptor between humans and mouse. Researchers also tried to deliver hepatitis C virus (Yu et al., 2014; Billerbeck et al., 2017) and hepatitis D virus (Suarez-Amaran et al., 2017) by HGD injection, and made efforts to establish corresponding infection mouse model. This may provide a better animal platform for mechanisms study and drug trial of the above hepatitis virus. Extensive genomic and biological studies of these viruses are thus enabled.

## Establishment of hepatocellular carcinoma models by HGD

The application of HGD in liver disease and hepatitis model described above have also raised interest in its application in liver cancer. Hepatocellular carcinoma (HCC) is one of digestive system malignant tumors, with a high mortality rate. People have developed various mouse models of HCC to explore the underlying mechanisms of HCC occurrence and metastasis, such as genetically engineered mouse (GEM) models of HCC (Newell et al., 2008). However, it is very expensive and time-consuming to generate a GEM model. Recently, HGD was applied to generate many models of HCC by over-expressing candidate oncogenes, coupled with the sleeping beauty transposon/transposase system (Ju et al., 2016). The sleeping beauty transposon /transposase vectors induced stable chromosomal integration and persistent transgene expression. HGD of the sleeping beauty transposon/transposase vector resulted in transpon DNA integrated in 5-6% hepatocytes of the mice (Yant et al., 2000). With this time-saving and economical approach, a variety of HCC models have been established very quickly, by activating oncogenes and/or silencing tumor suppressor genes. Tward et al reported that about three quarters of mice developed HCC in 1 month after HGD injection of transposon carrying Met and β-catenin gene (Tward et al., 2007). Besides, the researchers also tried CRISPR/Cas9-mediated gene editing, which allowed rapid occurrence of liver tumors, by regulating oncogenes or tumor suppressor genes expression in somatic cells (Liu et al., 2017).

The HGD of sleeping beauty transposon or CRISPR/Cas9 system could be applied not only to investigate potential oncogenic collaboration among various signaling pathways during HCC development, but also to test anti-HCC therapy preclinically. Sorafenib treatment in HCC models generated by HGD with sleeping beauty transposon effectively prolonged the median survival of mice with HCC, resembling that in HCC patients treated with sorefinib (Rudalska et al., 2014).

## Applications of HGD in vaccination

The hydrodynamic delivery method has also been employed for vaccination and active immunotherapy during viral infection. The hydrodynamic delivery of plasmid expressing herpes simplex virus type 1 glycoprotein (HSV-1gp) induced significant production of HSV-1 neutralizing antibody in mice. The HSV-1 gp gene vaccination protected mice from latent HSV infection (Cui et al., 2003). The vector encoding HBV core gene could elicit HBV immunization and control viral infection by inducing antigen expression in liver and blood via HGD (Deng et al., 2009). It was also demonstrated that hydrodynamic vaccination with HIV-1 envelope DNA elicited 40-fold HIV-1 envelope specific antibodies than other immunization methods (Raska et al., 2008). DNA vaccines targeted to the liver seemed resulting in enhanced immune response.

However, an adverse result was shown in LCMV vaccination. HGD of liver-specific LCMV-gp antigen resulted in lower memory CD8 T cell frequency than that in intramuscularly immunized mice, leading to reduced protection against lethal viral challenge. The results suggest limited priming in liver compared to peripheral tissues. In any case, HGD mediated vaccination is worth more extensive exploration in other chronic viral infections. The main applications of HGD in liver study were summarized in Table 1.

**Table 1:** Applications of HGD in liver study.

**Application**	**References**
**ALTERNATION OF GENE EXPRESSION IN LIVER MICROENVIRONMENT**	
Gene over-expression	Wang et al., 2001; Hong et al., 2003; Pan et al., 2004
Gene knockdown	Song et al., 2003; Hamar et al., 2004; Matsui et al., 2005
Simultaneous over-expression and knockdown	Geng et al., 2013
**ESTABLISHMENT OF MODELS**	
HBV	Yang et al., 2002; Huang et al., 2006
HCV	McCaffrey et al., 2002, 2003
HDV	Chang et al., 2001; Bordier et al., 2003
HCC	Tward et al., 2007; Keng et al., 2011; Ju et al., 2016
**VACCINATION**	
HSV-1	Cui et al., 2003
HBV	Deng et al., 2009
HIV	Raska et al., 2008
LCMV	Obeng-Adjei et al., 2013
**GENE THERAPY**	
Hepatitis	Anavi et al., 2013; Huang et al., 2013
Liver fibrosis	Chen S. W. et al., 2008; Kobayashi et al., 2016
**BASIC BIOLOGY STUDY**	
Liver-draining lymph nodes	Zheng et al., 2013

## Improvement of HGD for preclinical application

The above advantages of HGD in rodent studies have led researchers to consider its feasibility in clinical application. The first step is to reduce of the injected volume of HGD, while maintaining the hydrodynamic pressure for intracellular uptake. After all, an intravenous volume of 10% body weight is too large for human, which is equivalent to 5 L solution for a 50 kg adult. Cardiac congestion is well tolerated in mice, yet may not be safe in patients. At present, the common strategy used in large animals (dog, pig) is the replacement of systemic intravenous injection with specific delivery of target organs or sites via catheters. Based on this modification, the researchers combined hydrodynamic injection with a jugular vein catheter and then delivered the genes of interest to each hepatic lobular vein and hepatocytes (Kamimura et al., 2009, 2013, 2014). Human factor IV expression were highly expressed in the liver of dogs in this way (Yokoo et al., 2016). Although the gene delivery efficiency in these procedures is lower than HGD by tail vein in rodents, the results demonstrate the possibility of liver gene delivery with <1% of body weight volume (500 ml for a 50 kg adult), which might be clinically acceptable in human. To date, HGD has made significant progress in all aspects, which supported the clinical feasibility. The safety and side effects of this procedure in large animals are still under careful evaluation.

The accompanying step is the improvement and enrichment of substances for injection. The DNA and siRNA expressing vectors could be developed to be multi-functional, including liver-specific promoter insertion and simultaneous multi-gene expression. Many other molecules, such as pDNA-cationic liposome complexes (namely as lipoplex) (Templeton, 2002) or pDNA-cationic polymer complexes (polyplex) (Thomas and Klibanov, 2003), antisense or decoy oligonucleotides, chimeric DNA-RNA oligonucleotide duplex (Lai and Lien, 2001; Liang et al., 2002) or peptide nucleic acid (Ray and Norden, 2000) have been delivered by hydrodynamic injection (Table 2). Moreover, it has been demonstrated that macromolecules, including bovine serum albumin (BSA) and immunogloblin G (IgG), can be also effectively absorbed by the liver via hydrodynamic injection (Kobayashi et al., 2001).

**Table 2:** Substances entry into parenchymal cells by HGD methods.

**Delivery substances**	**References**
**NUCLEOTIDES**	
pDNA	Yang et al., 2001
^125^I-DNA	Zhang et al., 2004
Transposon	Yant et al., 2000; Tward et al., 2007
Antisense or decoy oligonucleotides	Tomita et al., 2003; Shoji and Nakashima, 2004
Chimeric DNA-RNA oligonucleotide duplex	Lai and Lien, 2001; Liang et al., 2002
Peptide nucleic acid	Ray and Norden, 2000
siRNA	Song et al., 2003; Zender et al., 2003
shRNA	Huang et al., 2013
**PROTEIN**	
Evans Blue	Zhang et al., 2004
Galactosidase	Zhang et al., 2004
**VIRUS**	
Adenovirus	Chen Q. et al., 2008; Huang et al., 2012, 2013; Zheng et al., 2013
Adeno-associated virus	O'Callaghan et al., 2017
Retrovirus	Wu et al., 2002
Lentivirus	Condiotti et al., 2004
Epstein-Barr virus	Stoll et al., 2001
**OTHER COMPOUNDS**	
pDNA-cationic liposome complexes	Templeton, 2002
pDNA-cationic polymer complexes	Thomas and Klibanov, 2003
Fluorescence-labeled beads	Kobayashi et al., 2004

## Conclusions and future prospects

As described above, hydrodynamic injection provides an effective approach to deliver DNA, RNA, protein and virus into parenchymal cells (especially hepatocytes). Besides the applications in the studies of liver diseases, HGD also contributes to basic understanding of liver biology. One of the examples is the discovery of liver draining lymph nodes in mice. At 24 and 48 h after hydrodynamic injection with enhanced green fluorescence protein (EGFP) expressing adenovirus through the tail vein, most of the cells expressing EGFP appeared in the portal and celiac lymph nodes near the liver, and a small number of EGFP expressing cells were present in the spleen and inguinal lymph nodes, which suggesting the portal and celiac lymph nodes may be the primary drainage lymph nodes in the liver of mouse (Zheng et al., 2013).

Although it has achieved a great success in rodent studies, more investigations are needed to advance its application in human. We anticipate that this could be achieved in the near future with the efforts on improving the function and diversity of substances for injection, as well as more precise control of injection routes and volumes which is a key step in clinical application. In general, HGD offers an useful tool for the study of liver and potentially other organs, and will continue to advance to benefit both basic and clinical research.

## Author contributions

MH and RS wrote the paper. QH and ZT instructed and supervised the writing.

### Conflict of interest statement

The authors declare that the research was conducted in the absence of any commercial or financial relationships that could be construed as a potential conflict of interest.
